# Distribution of Human Immunodeficiency Virus and Human T-Leukemia Virus Co-infection in Bahia, Brazil

**DOI:** 10.3389/fmed.2021.788176

**Published:** 2022-01-10

**Authors:** Felicidade Mota Pereira, Fred Luciano Neves Santos, Ângelo Antônio Oliveira Silva, Nathan Menezes Nascimento, Maria da Conceição Chagas Almeida, Roberto Perez Carreiro, Bernardo Galvão-Castro, Maria Fernanda Rios Grassi

**Affiliations:** ^1^Advanced Health Public Laboratory, Gonçalo Moniz Institute, Fundação Oswaldo Cruz - Bahia (FIOCRUZ-BA), Salvador, Brazil; ^2^Gonçalo Moniz Public Health Central Laboratory, Laboratório Central (LACEN), Salvador, Brazil; ^3^Bahiana School of Medicine and Public Health, Escola Bahiana de Medicina e Saúde Pública (EBMSP), Salvador, Brazil; ^4^Molecular Epidemiology and Biostatistics Laboratory, Gonçalo Moniz Institute, FIOCRUZ-BA, Salvador, Brazil; ^5^Center for Integration of Data and Health Knowledge, Centro de Integração de Dados e Conhecimentos para Saúde (CIDACS), Gonçalo Moniz Institute, FIOCRUZ-BA, Salvador, Brazil

**Keywords:** HTLV, HIV, coinfection, Bahia, epidemiology

## Abstract

Human Immunodeficiency Virus (HIV) and Human T-Leukemia Virus (HTLV) are retroviruses that share similar routes of transmission. In Brazil, the prevalence of HIV and HTLV varies according to geographic region. The state of Bahia, located in the Northeast region, is considered endemic for both retroviruses. The present study aimed to characterize the frequency of HIV/HTLV coinfection and evaluate the geographic distribution of coinfection throughout the state. This cross-sectional study was conducted at the state's Central Laboratory of Public Health (LACEN-BA) and included all samples from 2004 to 2013 submitted to serological testing for anti-HIV and anti-HTLV-1/2, screened by chemiluminescence/ELISA and confirmed by Western blot. Infection rates are expressed as the number of infected individuals per 100,000 inhabitants from each municipality. A total of 129,158 samples originating from 358/417 (85.8%) municipalities in Bahia were evaluated. HTLV was detected in 2.4% of the HIV-positive samples (*n* = 42) compared to 0.5% of those with negative HIV serology (*n* = 677) (OR: 4.65; CI: 3.39–6.37). HIV/HTLV coinfection was more frequent in women (69.0%); the median age of coinfected individuals was 47.2 years [interquartile range (IQR): 41.6–55.4 years]. In the 14/417 (3.4%) municipalities where at least one case of HIV/HTLV coinfection was detected, the overall HTLV coinfection rate in HIV-positive samples was 0.25 (range: 0.17–13.84) per 100,000 inhabitants. Most cases of HIV/HTLV-1 coinfection (21/37, 57%) were concentrated in the municipality of Salvador. Isolated instances (one or two cases) of HIV/HTLV-1 coinfection were distributed across municipalities known to be endemic for HTLV infection.

## Introduction

Human Immunodeficiency Virus (HIV) and Human T-Leukemia Virus (HTLV) are retroviruses that share similar routes of horizontal transmission, including unprotected sex, the sharing of needles, syringes, or other drug injection devices, as well as vertically from mother to child (1). HIV is divided into two main types: HIV-1, the most common type responsible for the AIDS pandemic, and HIV-2, which is relatively rare and less pathogenic (2). There are four types of HTLV. HTLV-1, the most frequently reported, causes both inflammatory and proliferative diseases, such as HTLV-1-associated myelopathy/tropical spastic paraparesis (HAM/TSP), adult T-cell leukemia (ATL), uveitis and infective dermatitis (3). HTLV-2 has not been clearly linked to disease development, and is mostly reported in Amerindians and injectable drug users (IDU) (4). HTLV-3 and HTLV-4, found in restricted areas in Western Africa, have not been associated with disease (5). It is estimated that around 35 million people are infected with HIV around the world (6), while HTLV affects ~10 million people (3). Rates of HIV/HTLV coinfection vary worldwide, with higher rates found in larger metropolitan regions throughout the Americas, Europe, and Africa (7). Both viruses are endemic in Brazil, where it is estimated that over one million individuals live with HIV, while around 800,000 live with HTLV-1 (3, 8).

In Brazil, the prevalence of HIV and HTLV vary according to geographic region, with higher rates of HIV found in the Southeast and South regions, while HTLV is more frequently detected in the North and Northeast regions (8, 9). Studies evaluating HTLV in HIV-infected populations from several regions of Brazil have demonstrated a prevalence varying from 1.5 to 10.9% (10–16).

Coinfection with HTLV may interfere with the outcome of HIV infection. A Brazilian study showed that HIV/HTLV co-infected patients presented shorter survival times compared to HIV-monoinfected individuals (17). Infection with HTLV-1 induces the pronounced activation of CD4^+^ T-lymphocytes, which may provoke dysfunction in this cellular subset, possibly favoring the appearance of opportunistic diseases, the progression to AIDS and increased mortality in co-infected patients (18). On the other hand, it has been suggested that HIV/HTLV-2 coinfection may delay the progression to AIDS and prolong survival (19).

In Salvador, the capital of the state of Bahia, located in northeastern Brazil, the prevalence of HIV and HTLV-1 in the general population have been estimated at 0.55 and 1.8%, respectively (20, 21). Since Bahia is considered endemic for both retroviruses, the present study aimed to better characterize the frequency of HIV/HTLV coinfection and evaluate the geographic distribution of coinfection throughout the state of Bahia.

## Materials and Methods

### Ethics Statement

This study received ethical approval from the Institutional Review Board (IRB) for Human Research at the Goncalo Moniz Institute, Oswaldo Cruz Foundation (IGM-FIOCRUZ), Salvador, Bahia-Brazil (CAAE number 22478813.7.0000.0040). All identifiable patient information was anonymized to maintain confidentiality, thereby avoiding the need for individual verbal or written consent.

### Study Area

**Figure 1:**
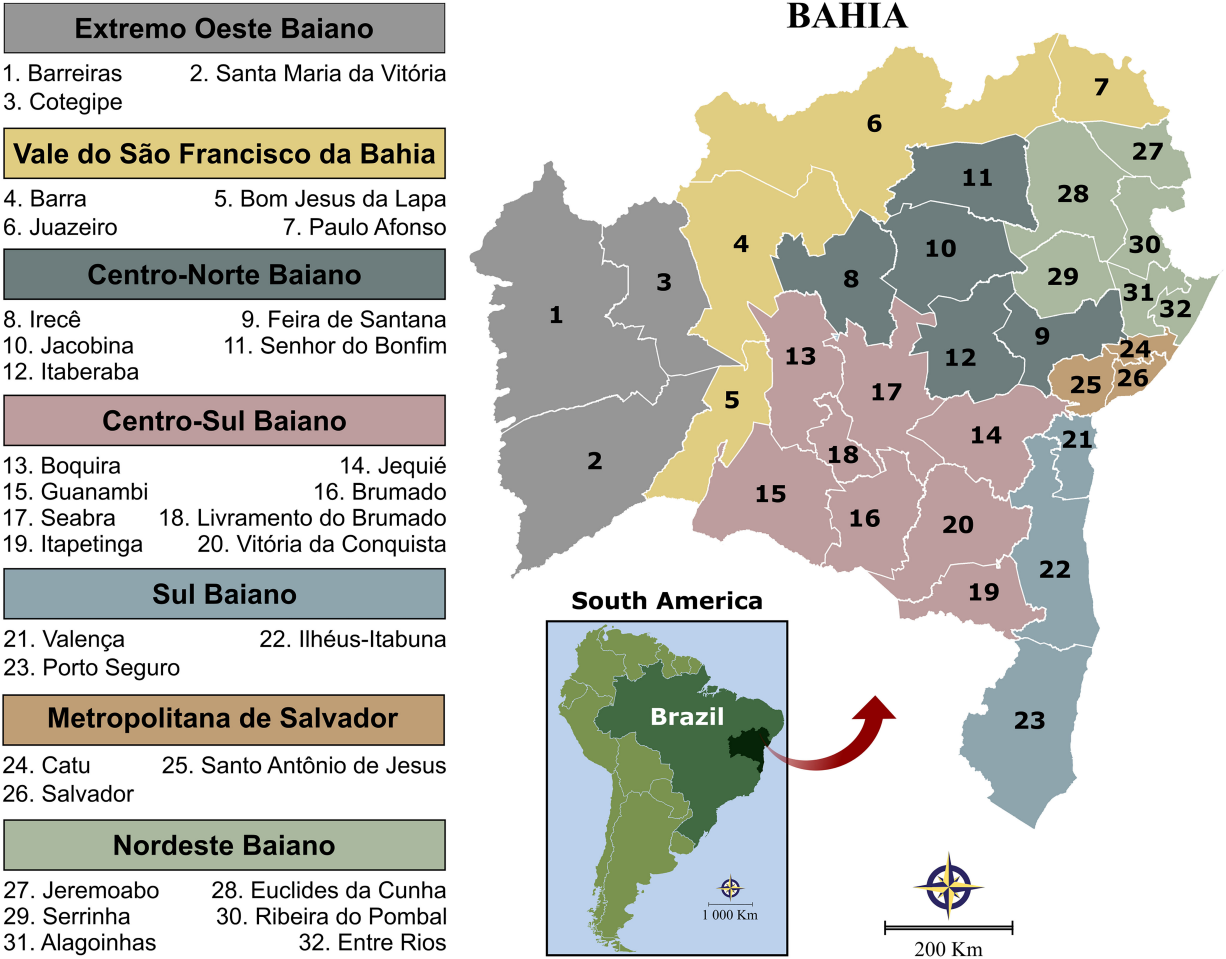
Illustration of the state of Bahia's seven mesoregions and 32 microregions, as determined by IBGE to more accurately reflect economic and social similarities among the 417 municipalities. Digital maps in the public domain were obtained from the IBGE cartographic database in shapefile format (.shp), which was subsequently reformatted and analyzed using QGIS version 3.10 (Geographic Information System, Open-Source Geospatial Foundation Project. http://qgis.osgeo.org).

All samples evaluated in this study originated from municipalities in the state of Bahia, Brazil. According the 2015 Brazilian national census, the total state population was 15,203,934 inhabitants (representing the fourth-largest state in terms of population size), with an overall density of 27 inhabitants per km^2^ (565,733 km^2^, the fifth-largest state in terms of area) (http://www.ibge.gov.br). The state is comprised of 417 municipalities, which have been grouped into 32 microregions and seven mesoregions by the Brazilian National Institute of Geography and Statistics (IBGE) in accordance with the economic and social similarities (Figure 1).

### Study Design

A cross-sectional study was conducted using data obtained from the Central Laboratory of Public Health of Bahia (LACEN-BA), responsible for infectious disease surveillance via laboratory analysis throughout the state. This laboratory's target population is mainly comprised of blood donors, pregnant women and individuals exhibiting symptoms of infectious disease, whose blood samples are referred by blood banks, prenatal physicians or clinicians working in the public health system. All individuals who were tested for both HIV and HTLV and had one or more serological samples submitted for analysis at LACEN-BA between 2004 and 2013, were investigated. Samples were classified as HTLV-1, HTLV-2 or co-infection with HTLV-1/2—the latter indicating that WB testing bands were positive for both HTLV-1 and HTLV-2 infection accordingly to the manufacturer's instructions. To avoid multiplicity, each individual's most recent HIV and HTLV serological results were considered. Only individuals with HIV and HTLV positivity confirmed by Western blot were included.

### Laboratory Testing

HIV serology was performed from 2004 to 2008 using an Elecsys HIV combi PT kit (Roche, Basel, Switzerland), which offers 100% sensitivity and 99.88% specificity; in 2009, the Architect HIV Ag/Ab Combo (ABBOTT, Germany), with 100% sensitivity and 99.5% specificity, was adopted. After detection of HIV seropositivity, confirmatory Western blot (HIV Blot 2.2, MP Diagnostics, Singapore) was performed for all samples. Serological testing for HTLV was performed by ELISA from 2004 to 2008 using the Murex HTLV-1/2 kit (DiaSorin S.p.A., Dartford, United Kingdom) with a sensitivity of 100% and a specificity of 99.94%, whereas from 2009 to 2010 the anti-HTLV-1/2 kit Sym Solution (Symbiosis Diagnostica LTDA, Leme, Brazil) was used (100% sensitivity, 99% specificity). In 2011, a microparticle CLIA chemiluminescence assay (Architect rHTLV-1/2, Abbott Diagnostics Division, Wiesbaden, Germany) was used, offering a sensitivity of 100% and a specificity of 99.5%. If HTLV seropositivity was detected, all samples were submitted to confirmatory Western blotting (HTLV Blot 2.4, Genelabs Diagnostics R, Singapore).

### Data Analysis

Data were extracted from clinical records at LACEN using the SMART LAB laboratory manager, then transformed using Talend Open Studio Data Integrator software to integrate serologic and confirmatory test results on an annual basis. The analyzed database was formatted using high volume extract, transformation and load throughput (ETL). Subsequently, a CVS table (Comma, separated values) was generated for validation using the R package and extant analysis by the STATA v13.0 software. Absolute and relative frequencies were calculated for the following categorical variables: age group (0–10, 11–19, 20–29, 30–39, 40–49, 50–59, 60–69, and ≥70 years), sex and serological test result. All extracted data were grouped according to municipality and/or microregion for improved accuracy with regard to differences among regions. Digital maps obtained from the IBGE cartographic database in shapefile format (.shp) were subsequently reformatted and analyzed using QGIS software version 3.10 (Geographic Information System, Open-Source Geospatial Foundation Project; freely available at: http://qgis.osgeo.org) to determine the geographical distribution of HIV and/or HTLV in the state of Bahia. Three-year moving averages were calculated between 2004 and 2013 to construct spatial distribution maps and to minimize the effects of random fluctuations. Infection rates are expressed as the number of infected individuals per 100,000 inhabitant.

## Results

**Figure 2:**
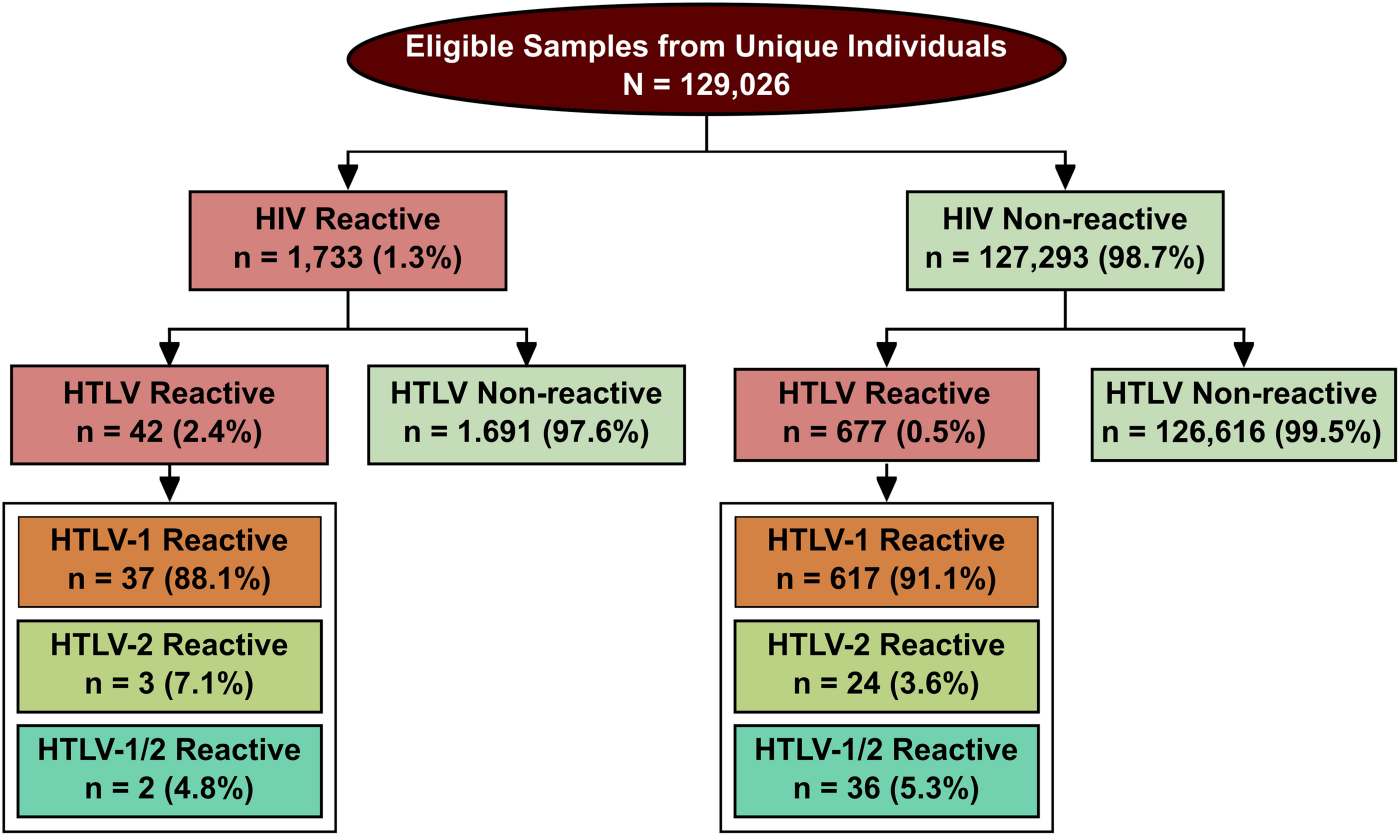
Flowchart describing study design and results of HIV and HTLV infection status in the studied population. All samples determined positive by ELISA screening were then confirmed by WB.

From 2004 to 2013, a total of 129,158 individuals were submitted to serological testing for both anti-HIV and anti-HTLV-1/2 (Figure 2). The median age of the studied population was 29.5 years [interquartile range (IQR): 23.8–36.9 years], and the female: male ratio was 10:1. The global prevalence of HIV was estimated at 1.3% (*n* = 1,733) in the samples analyzed, while the global prevalence of HTLV was 0.56% (*n* = 719). HTLV was detected in 2.4% of the HIV-positive individuals (*n* = 42) compared to 0.5% of samples with negative HIV serology (*n* = 677) (OR: 4.65; CI: 3.39–6.37) (Figure 2).

**Figure 3:**
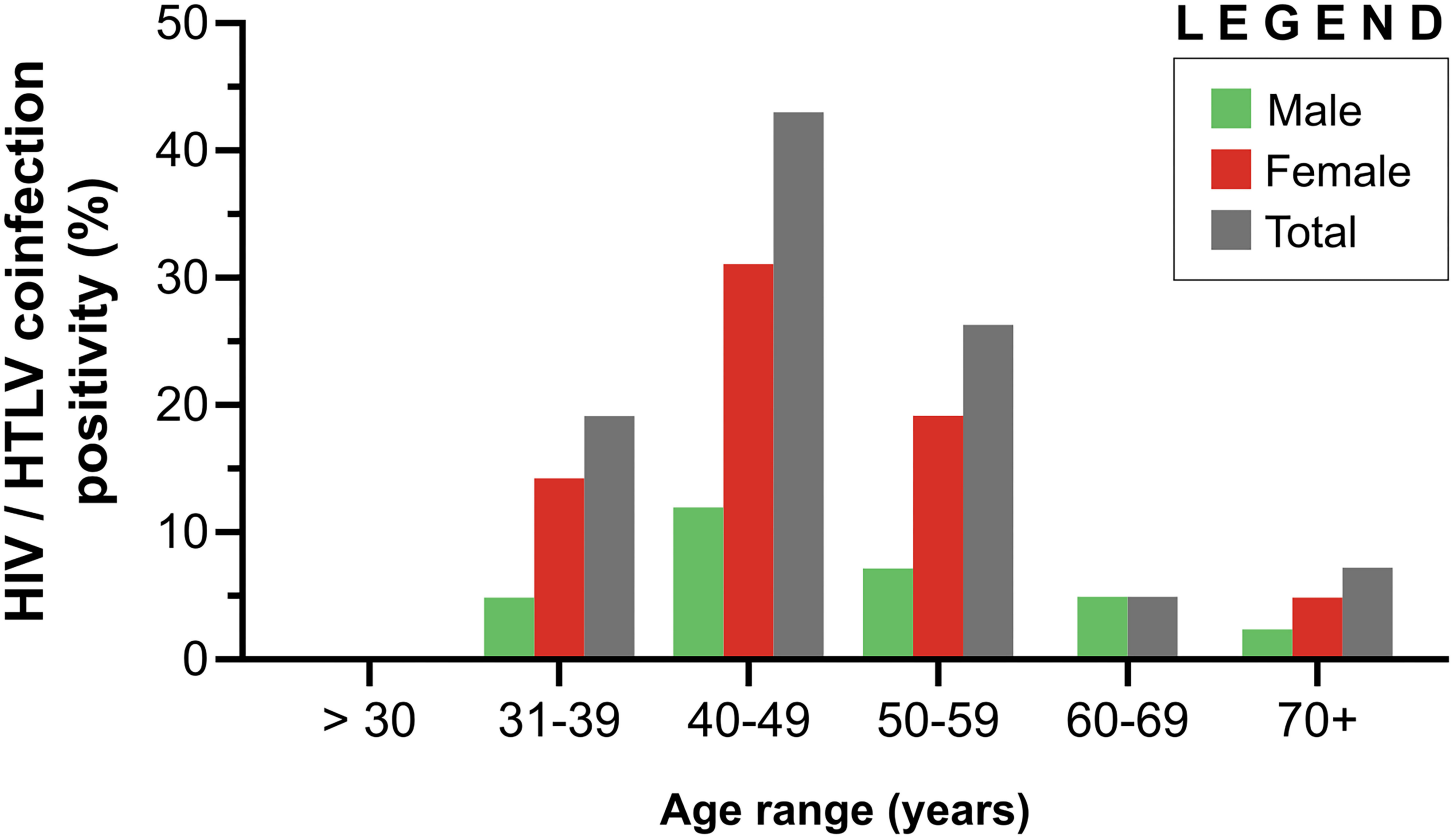
Distribution of HIV/HTLV coinfection according to age among 29 females and 13 males in Bahia, Brazil (2004–2013).

HIV/HTLV coinfection (*n* = 42) was more frequently found in women (69.0%) compared to men (31.0%) (*p* < 0.0001) (Table 1). The median age of the 42 HIV/HTLV cases was 47.2 years [interquartile range (IQR): 41.6–55.4 years]. With regard to age distribution, 42.9% of these individuals were aged between 40 and 49 years, followed by 50–59 years (26.2%), 31–39 years (19.0%), over 69 years (7.1%) and 60–69 years (4.8%). No cases of co-infection were detected in individuals aged 30 years or less (Figure 3).

**Table 1:** Profile of HTLV/HIV coinfection in municipalities throughout Bahia, Brazil (2004–2013).

**Municipality**	Population^*^	**Microregion**	**# Samples (%Female)**	**# Co-infection cases**	**Age**	**%Female**	Co-infection rate^**^
**HTLV-1**
Barreiras	136,741	Barreiras	89 (56.2)	1	48	100	0.73
Feira de Santana	588,102	Feira de Santana	484 (65.3)	1	48	100	0.17
Iaçu	28,609	Itaberaba	591 (94.9)	1	56	100	3.50
Ipirá	61,818	Feira de Santana	484 (65.3)	1	56	100	1.62
Itagi	14,190	Jequié	200 (89.0)	1	48	100	7.05
Itamaraju	67,660	Porto Seguro	5,655 (80.0)	2	54	100	2.96
Lauro de Freitas	154,976	M. de Salvador^a^	2,095 (88.1)	2	46	100	1.29
Muniz Ferreira	7,225	Sto A. de Jesus^b^	7 (57.1)	1	33	0	13.84
Porto Seguro	121,678	Porto Seguro	255 (87.1)	2	56	100	1.64
Salvador	2,973,395	M. de Salvador^b^	24,330 (83.3)	21	49	61.9	0.71
Santo Amaro	58,031	Sto A. de Jesus^b^	153 (91.5)	1	39	100	1.72
Simões Filho	115,656	Salvador	1,719 (91.5)	1	44	100	0.86
Teixeira de Freitas	124,644	Porto Seguro	1,286 (91.5)	1	45	0	0.80
Una	24,969	Ilhéus-Itabuna	353 (87.5)	1	38	100	4.01
**HTLV-2**
Paramirim	26,670	L. do Brumado^c^	248 (98.8)	1	66	0	4.80
Salvador	2,973,395	M. de Salvador	24,330 (83.3)	2	58	50	0.07
**HTLV-1/2 coinfection**
Salvador	2,973,395	M. de Salvador	24,330 (83.3)	2	46	50	0.07

*Mean age (years)*;

* *Mean population size from 2008 to 2009 (www.ibge.gov.br);*

** *No. of cases per 100,000 inhabitants*.

a *Metropolitana de Salvador;*

b *Santo Antônio de Jesus;*

c *Livramento do Brumado*.

In the 42 cases of coinfection, WB identified 37 (75.5%) samples as positive for HTLV-1 (prevalence ~0.03%), 3 (6.1%) for HTLV-2 (prevalence ~0.002%) and 2 (4.1%) were positive for both HTLV-1 and HTLV-2 (prevalence ~0.002%) (Figure 2). Considering those individuals who tested negative for HIV but were positive for HTLV (*n* = 677), 617 of these samples were positive for HTLV-1 (91.1%), 24 for HTLV-2 (3.6%) and 36 (5.3%) were positive for both HTLV-1 and HTLV-2.

**Figure 4:**
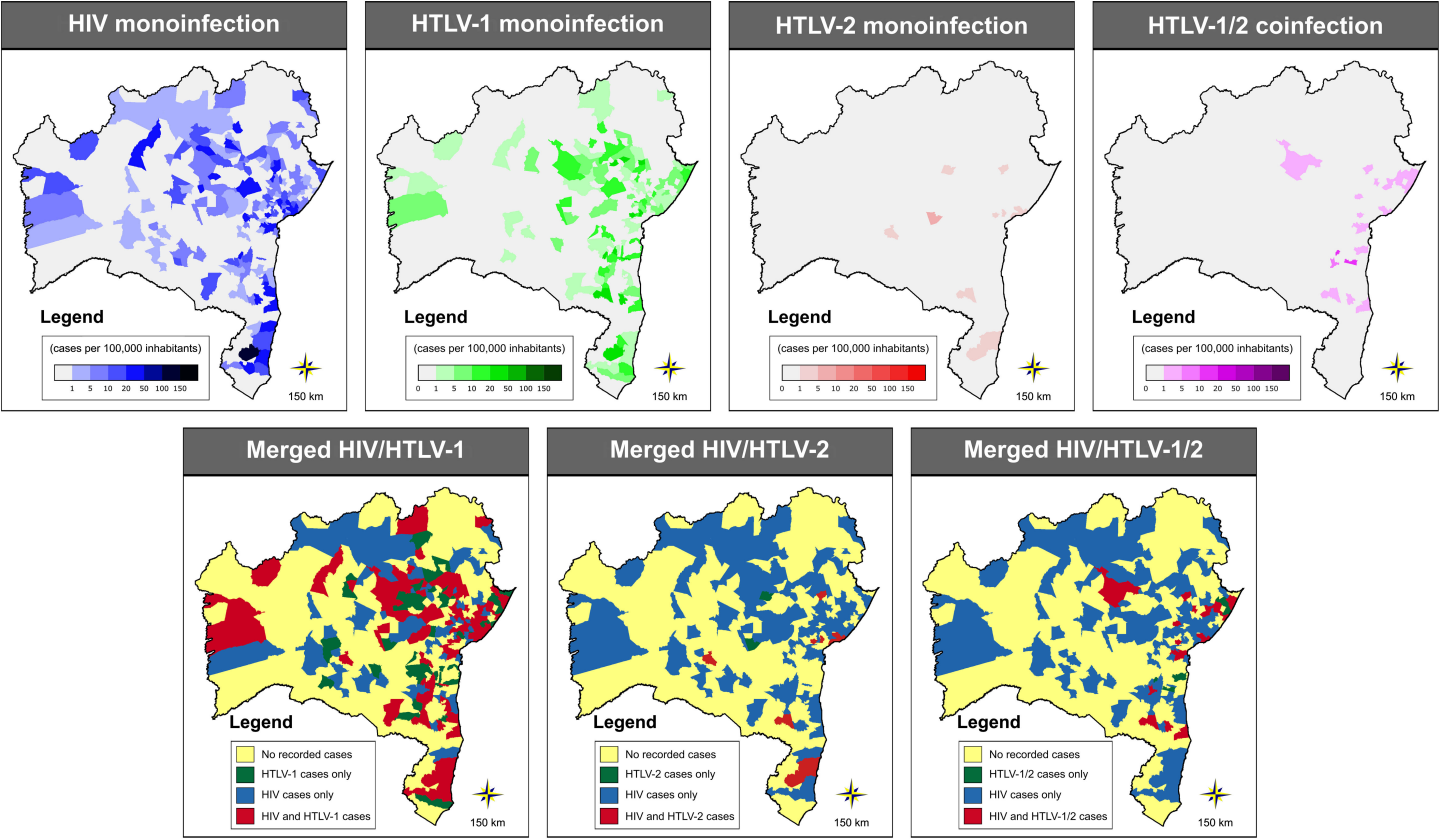
Geographic distribution of cases of HIV-1, HTLV-1, HTLV-2 and HTLV-1/2, as well as merged graphs detailing the distribution of two or more of these infections, throughout the municipalities in the state of Bahia (2004–2013). Public domain digital maps were obtained in shapefile format (.shp), subsequently reformatted and analyzed using QGIS version 3.10 (Geographic Information System, Open-Source Geospatial Foundation Project. http://qgis.osgeo.org).

Out of 417 municipalities in the state of Bahia, 358 (85.8%) sent samples to LACEN at some time during the study period. At least one case of HIV was reported in 155 (37.2%) municipalities, while HTLV-1, HTLV-2 and HTLV-1/2 coinfection were detected in 121 (29.0%), 13 (3.1%) and one (0.2%) of the municipalities, respectively (Figure 4).

**Figure 5:**
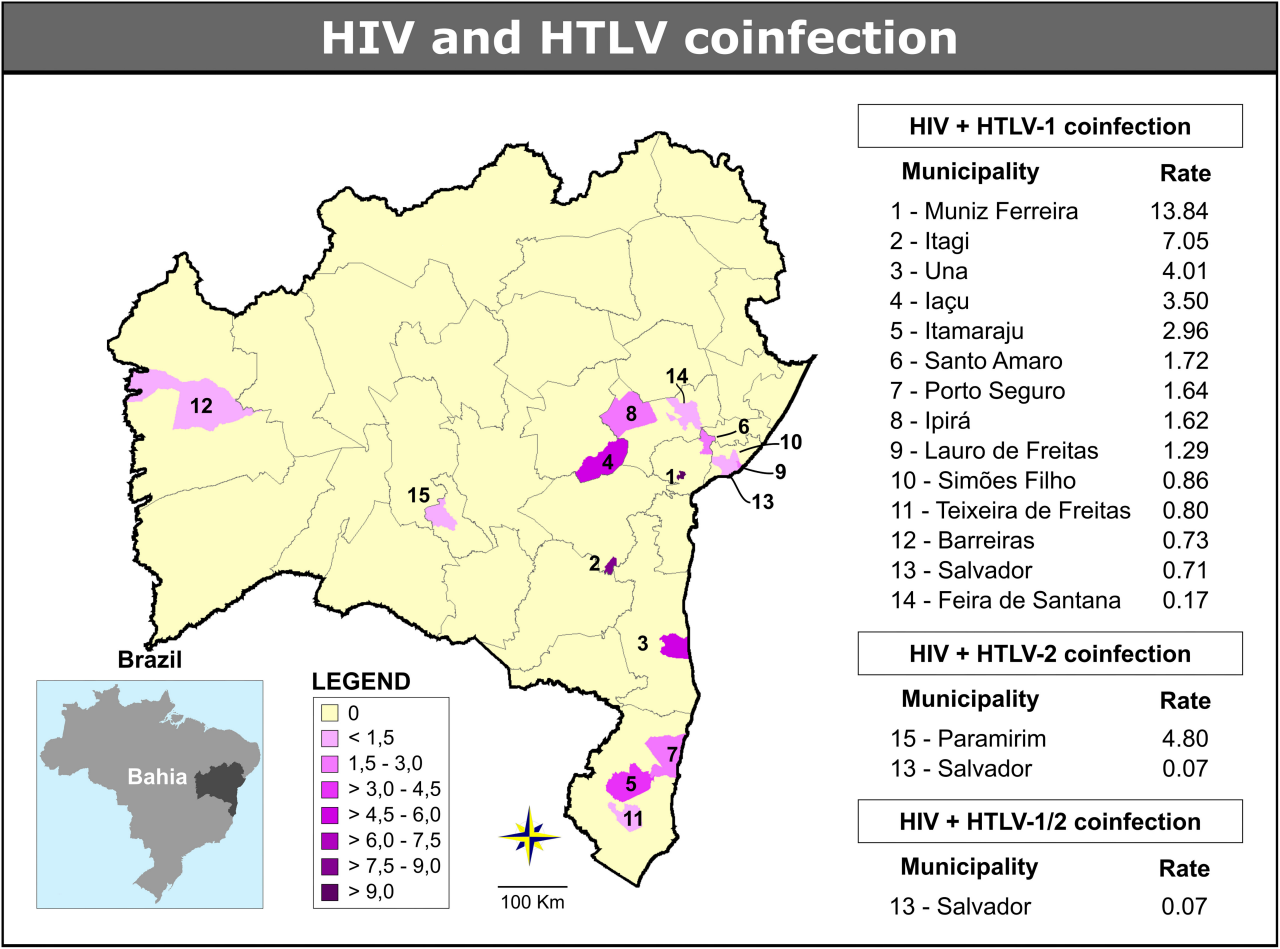
Geographic distribution of cases of HIV/HTLV-1, HIV/HTLV-2, and HIV/HTLV-1/2 coinfection among municipalities in the state of Bahia (2004–2013). Microregions are delimited by gray lines. Public domain digital maps were obtained in shapefile format (.shp), subsequently reformatted and analyzed using QGIS version 3.10 (Geographic Information System, Open-Source Geospatial Foundation Project. http://qgis.osgeo.org).

In all, 14 (3.4%) municipalities presented at least one case of HIV/HTLV-1 coinfection, with an overall rate estimated at 0.25 per 100,000 inhabitants (range: 0.17–13.84/100,000 inhabitants) (Figure 5). The highest rates were found in the following five municipalities: Muniz Ferreira (13.84 cases per 100.000 inhabitants), Itagi (7.05 cases per 100.000 inhabitants), Una (4.01 cases per 100.000 inhabitants), Iaçu (3.50 cases per 100.000 inhabitants) and Itamaraju (2.96 cases per 100.000 inhabitants) (Table 1; Figure 5). The majority of cases of HIV/HTLV-1 coinfection (21/37, 57%) were concentrated in the municipality of Salvador. Isolated instances (one or two cases) of HIV/HTLV-1 coinfection were distributed in municipalities across eight microregions (Table 1), concentrated mainly in the Salvador (*n* = 21), Porto Seguro (*n* = 5), and Feira de Santana (*n* = 2) microregions.

The global rate of HIV/HTLV-2 coinfection was estimated at 0.02 per 100,000 inhabitants (range: 0.07–4.80/100,000 inhabitants) and identified in two municipalities: one case in Paramirim (4.80 cases per 100.000 inhabitants) and two cases in Salvador (0.07 case per 100.000 inhabitants) (Table 1; Figure 5).

The two cases of triple infection were found in the Salvador microregion (0.07 cases per 100,000 inhabitants) (Table 1; Figure 5). The overall rate of HIV/HTLV-1/HTLV-2 co-infection was estimated at 0.01 per 100,000 inhabitants.

## Discussion

Our work represents the first large-scale study to evaluate HIV/HTLV coinfection in the state of Bahia (Brazil), an area considered endemic for both infections (22, 23). While HIV and HTLV monoinfection was detected throughout most of the state's microregions, coinfection occurred sporadically in 15 out of 417 (3.4%) of the state's municipalities, leading to an overall rate of 0.25 per 100,000 inhabitants. A previous study reported widespread HTLV infection throughout the state of Bahia at an overall rate of 14.4/100,000 inhabitants. Many of the municipalities in which HIV/HTLV coinfection cases were presently identified are in microregions where clusters of HTLV infection were previously reported, such as Salvador, Barreiras and Porto Seguro (24). Although the prevalence of HIV infection remains unknown in the state of Bahia, the global rate of AIDS detection in the state was 12.9/100,000 inhabitants from 2015 to 2019. The cities of Salvador, Ilhéus, Teixeira de Freitas, Porto Seguro and Feira de Santana ranked among the top 100 Brazilian cities with the highest rates of AIDS per 100,000 inhabitants (range: 17.7–27.9/100,000 inhabitants) (23). The microregions in which HIV/HTLV coinfection was detected share similar characteristics with respect to economic activity, consisting mainly of commercial, service, tourism, and industrial economies. As the municipality of Salvador, the state's capital, is one of the most endemic areas for HTLV-1 in Brazil (9, 25), it was expected that the largest absolute number of coinfected individuals would be found in this municipality. Population-based studies conducted in this municipality have reported that ~0.5 and 1.5% of residents harbor HIV and HTLV-1 infection, respectively (20, 21). The Barreiras microregion is one of the most developed in the state, with abundant agribusiness and commercial activity. Due to its proximity to Brasília, the federal district, as well as its importance as an agricultural hub, the area receives regular influxes of workers as well as many visitors. In the Porto Seguro microregion, located in the southernmost region of the state, tourism and commercial activities are essential to the local economy. A network of federal highways connecting Bahia to different parts of the country runs through the Feira de Santana and Itaberaba microregions; these areas are important for several regional industries and generate significant commercial activity. All regions where HIV/HTLV coinfection was identified are important to the state's economy, which suggests while that the circulation of both viruses is relevant, co-infection is dependent on multiple factors, notably risk behaviors, such as unprotected sexual activity. It is therefore expected that there is a higher potential for exposure to viruses in these areas due to tourism and/or economic activity, which encourages populational transience (26).

In the present study, 2.4% of HIV-infected individuals were found to be coinfected with HTLV, in contrast to just 0.5% among those not infected with HIV. This translates to an almost five times greater risk of HTLV infection in individuals infected with HIV. Several studies have demonstrated that individuals infected by HIV have an increased risk for HTLV infection than the general population. In Brazil, the reported prevalence of HTLV infection in HIV-infected individuals ranges between 1.6% and 10.9% (11–16); lower rates have been observed in blood donors (0.006%) (27) and pregnant women (2.7–3.4%) (28, 29). Similar to the present results, most cases of HIV/HTLV coinfection reported in Brazil are associated with HTLV-1 (14, 28, 30), except in Belém municipality, located in the Amazon region, in Northern Brazil, where HTLV-2 predominates among co-infected (10). Notably, HIV/HTLV coinfection in the US and Europe has been predominantly associated with HTLV-2, primarily due to injecting drug use (7, 31, 32). In addition, since HTLV-1 is considered endemic in Brazil, notably in Bahia, it follows that higher rates of HIV/HTLV-1 coinfection would be detected compared to HIV/HTLV-2 (22, 24). Furthermore, sexual contact has been shown to be a more relevant route of transmission for HTLV in the population of Salvador, where HTLV-1 is highly prevalent, which lends further support to the comparatively higher rate of HIV/HTLV-1 coinfection observed herein (21). Considering that the prevalence of HTLV-1 increases with age, this may explain the discrepancy between the median age of the total cohort (29.5 years) and that of the coinfected individuals (42 years) (22).

Studies have associated HIV/HTLV coinfection with risk factors that include previous blood transfusion, intravenous drug use and sexual contact with multiple partners (7, 14, 33, 34). A previous study conducted in Salvador demonstrated a 22.2% rate of HIV/HTLV-1 and 10.6% of HTLV/HTLV-2 coinfection in injectable drug users in contrast to the frequency found herein (2.4%) (35).The low prevalence of coinfection of HIV/HTLV found in the present study may be partially explained by the Brazilian response to the HIV/AIDS epidemic among injecting drug users, including the establishment of harm reduction programs that provide sterile needles and syringes (36). Another important factor that likely contributed to the lower rate of coinfections is changing patterns of drug use in Bahia, i.e., the replacement of injecting drugs with inhalants (37).

The main limitation of the present study was the use of non-random sampling, which resulted in a predominance of females. In the state of Bahia, serological HTLV screening for pregnant women was become compulsory in 2011, which surely contributed to a higher proportion of women in the samples analyzed. Therefore, gender imbalance may have impacted the higher frequency of HIV/HTLV coinfection in women observed herein. Another limitation was the absence of information collected on risk factors for HIV/HTLV coinfection. However, with respect to the representativeness of the municipalities, ~86% of the state municipalities were evaluated throughout the study.

In conclusion, HIV/HTLV coinfection in Bahia, an area endemic for both viruses, can be considered a rare event, detected in just 2.4% of all HIV-infected individuals evaluated and in 3.4% of the municipalities throughout the state. The areas with higher numbers of coinfection cases were those considered hotpots for HTLV and HIV, representing important economic or tourist centers in the state. The identification of relevant risk factors associated with HTLV/HIV coinfection can lead to efficacious actions in a variety of epidemiological contexts specific to each affected region. It is our hope that these findings will provide support for the implementation of preventive measures to contain the spread of these viruses, especially in areas where higher rates of HTLV/HIV coinfection were described.

## Data Availability Statement

The raw data supporting the conclusions of this article will be made available by the authors, without undue reservation.

## Ethics Statement

The studies involving human participants were reviewed and approved by Institutional Review Board (IRB) for Human Research at the Goncalo Moniz Institute, Oswaldo Cruz Foundation (IGM-FIOCRUZ). Written informed consent from the participants' legal guardian/next of kin was not required to participate in this study in accordance with the national legislation and the institutional requirements.

## Author Contributions

FP, FS, BG-C, and MR: conceptualization. FP, FS, ÂS, NN, MA, RC, and MR: data curation. FP, FS, MA, RC, and MR: formal analysis and methodology. All authors involved in investigation, writing—original draft, review and editing, contributed to the article, and approved the submitted version.

## Funding

This work was supported by the Coordination of Superior Level Staff Improvement Coordenação de Aperfeiçoamento de Pessoal de Nível Superior (CAPES) finance code 001, National Council for Scientific and Technological Development (Conselho Nacional de Desenvolvimento Científico e Tecnológico-CNPq), National Foundation for the Development of Higher Education (Fundação Nacional para o Desenvolvimento do Ensino Superior-FUNDADESP), and Foundation for Research Support of the State of Bahia (Fundação de Amparo à Pesquisa do Estado da Bahia-FAPESB). Maria Fernanda Rios Grassi, Bernardo Galvão-Castro, and Fred Luciano Neves Santos are research fellows of CNPq (process no. 304811/2017-3, 311054/2014-5, and 309263/2020-4, respectively).

## Conflict of Interest

The authors declare that the research was conducted in the absence of any commercial or financial relationships that could be construed as a potential conflict of interest.

## Publisher's Note

All claims expressed in this article are solely those of the authors and do not necessarily represent those of their affiliated organizations, or those of the publisher, the editors and the reviewers. Any product that may be evaluated in this article, or claim that may be made by its manufacturer, is not guaranteed or endorsed by the publisher.
